# Preparation of Self-supporting Bagasse Cellulose Nanofibrils Hydrogels Induced by Zinc Ions

**DOI:** 10.3390/nano8100800

**Published:** 2018-10-08

**Authors:** Peng Lu, Ren Liu, Xin Liu, Min Wu

**Affiliations:** 1 Institute of Light Industry and Food Engineering, Guangxi Key Laboratory of Clean Pulp and Papermaking and Pollution Control, Guangxi University, Nanning 530004, China; lupeng@gxu.edu.cn (P.L.); RL@st.gxu.edu.cn (R.L.); 1405100409@st.gxu.edu.cn (X.L.); 2 State Key Laboratory of Pulp and Paper Engineering, South China University of Technology, Guangzhou 510640, China; 3 Key Laboratory of Pulp and Paper Science & Technology of Ministry of Education/Shandong Province, Qilu University of Technology, Jinan 250353, China

**Keywords:** hydrogel, cellulose nanofibrils, zinc ions, ionic cross-linking, bagasse cellulose fiber

## Abstract

Cellulose hydrogels are often prepared from native cellulose through a direct cellulose dissolution approach that often involves tedious process and solvent recovery problems. A self-supporting cellulose hydrogel was prepared by gelation of the TEMPO-oxidized bagasse cellulose nanofibrils (CNF) triggered by strong crosslinking between carboxylate groups and Zn^2+^. TEMPO process was used to generate negatively charged carboxylate groups on CNF surface to provide a high binding capability to Zn^2+^. Three TEMPO-oxidized CNFs of different carboxylate contents were prepared and characterized. TEM and AFM microscopes suggested that the sizes of CNFs were fined down and carboxylated cellulose nanofibrils (TOCNFs) of 5–10 nm wide, 200–500 nm long, and carboxylate contents 0.73–1.29 mmol/g were obtained. The final structures and compressive strength of hydrogels were primarily influenced by interfibril Zn^2+^-carboxylate interactions, following the order of TOCNFs concentration > content of carboxylate groups > concentration of zinc ions. A CO_2_ sensitive self-supporting cellulose hydrogel was developed as a colorimetric indicator of food spoilage for intelligent food packaging applications.

## 1. Introduction

Cellulose nanofibrils (CNFs) are novel nanomaterials prepared from natural cellulose fibers. CNFs contain micrometer-long entangled fibrils with a high aspect ratio of 4–20 nm in width and 500–2000 nm in length [1]. Currently, CNFs are mainly produced from cellulosic fibers by mechanical treatments (e.g., homogenization, grinding, and milling), enzyme-assisted mechanical treatments, chemical treatments (e.g., TEMPO oxidation), and a combination of chemical and mechanical treatments [2,3,4]. CNFs are of great interest for various applications relevant to the fields of material science and biomedical engineering due to its excellent mechanical properties, good biocompatibility, and tailorable surface chemistry [2]. 

Hydrogels are a class of three-dimensional networks formed by hydrophilic polymer chains embedded in a water-rich environment [5]. Structured hydrogel materials derived from cellulose are of increasing interest for biomedical, cosmetic, and other applications where biocompatibility and biodegradability are required [6,7,8,9]. Cellulose has many hydroxyl groups that can easily form hydrogen bonding linked networks, thus easily forming hydrogels with fascinating structures and properties [10]. Cellulose hydrogels are generally prepared from native cellulose through direct cellulose dissolution, using solvents such as N-methylmorpholine-N-oxide (NMMO), ionic liquids (ILs), and alkali/urea aqueous systems [6,11]. However, cellulose dissolution approaches often require multiple processing steps and also involve solvent recovery problems. 

Recently, stable cellulose nanofibrils based hydrogels have been fabricated by cross-linking surface carboxylate groups of CNFs with selected divalent and trivalent metal cations (Ca^2+^, Cu^2+^, Al^3+^, and Fe^3+^) [10,12,13,14]. Moreover, the mechanical properties of the CNFs hydrogels can be tailored by altering the polymer chemistry, cross-link density, and metal salt solution. Therefore, structuring cellulose hydrogel through a simple ionic-crosslinking approach opens new possibilities for preparing functionalized CNFs based nanomaterials to meet a variety of potential applications [15,16,17]. However, previous studies have primarily focused on preparing functional CNFs hydrogels with different metal species, but few reports discussed the influences of CNFs surface structure on the properties of CNFs hydrogels [18,19,20]. In fact, the CNFs hydrogels are formed because of reducing electrostatic repulsion between the surface carboxylate groups and partially assemble of adjacent CNFs [21]. The amount of carboxylate groups and mechanical properties of individual nanofibrils are believed to be related to the physical structure of CNFs network. Therefore, we propose that the characteristics of individual CNF (i.e., aspect ratios, surface chemistry, and crystal structure) may affect the integration mode of the CNFs as well as corresponding gels structures and properties.

The objective of this study is to prepare a self-supporting cellulose hydrogel through gelation of the CNFs aqueous dispersion with Zn^2+^. Particularly, CNFs prepared from bagasse instead of wood fibers were used, and to the best of our knowledge, no work has been published on the use of bagasse agricultural residue in this area. The characteristics of CNFs (i.e., morphology, surface charges, and carboxyl group contents), concentrations of CNFs as well as zinc ions in the aqueous system are proposed to be influencing factors to the strength of CNFs hydrogels and were investigated. In addition, a CNFs hydrogel-based CO_2_ sensitive indicator was first developed to explore the potential application of CNFs hydrogels in intelligent packaging. 

## 2. Materials and Methods 

### 2.1. Chemicals and Materials

Bleached bagasse pulp was obtained from Guangxi Guitang Pulp Mill (Guiguang, Guangxi, China). 2,2,6,6-tetramethylpiperidine-1-oxyl (TEMPO), sodium bromide, and sodium hypochlorite solution (available chlorine content 112.240 g/L) were purchased from Aladdin^®^ (Shanghai, China). Water was purified with the Milli-Q^®^ HX 7000 (Millipore, Burlington, MA, USA). Bromothymol blue (Aladdin^®^, Shanghai, China) and methyl red (Aladdin^®^, Shanghai, China) were used to dye the hydrogel for indicator preparation. Other chemical reagents used in this research were of chemical grade without any purification. 

### 2.2. Preparation and Characterization of CNFs

The bagasse pulp was diluted with water to a concentration of 2% (*w*/*w*) and disintegrated for 30 min. The pulp was then submitted to grinding using Masuko grinding mill (MKZA10-15J, Kawaguchi, Japan), with the gap between the two discs 4 points below the zero. The pulp was submitted to grounding for 11 passes, and then the wet sample was collected and stored at 4 °C until analysis. The solid content of as-prepared CNF slurry was calculated to be 2.48% (*w*/*w*).

The mean diameter and zeta potential of prepared CNF were determined by a Malvern Mastersizer (ZS90X, Malvern Instruments Limited, Worcestershire, UK) from 3 replicate measurements. 

The morphology of CNFs was characterized by transmission electron microscopy (TEM). Specifically, a drop of CNFs water suspension (0.008% *w*/*v*) was deposited on a carbon-coated electron microscope grid and then stained by 20 μL phosphotungstic acid water solution (1.5% *w*/*w*), followed by drying at room temperature before observation. The grid was observed under the standard conditions using a TEM (HT7700, Hitachi High-Tech, Tokyo, Japan) operating at 100 kV.

Atomic force microscopy (AFM) was also used to characterize the CNFs at a 1 μm × 1 μm scan size. Innovative scanning probe microscopy (SPM) with an AFM 5000II controller (Hitachi High-Tech, Tokyo, Japan) in a tapping mode was used with a silicon probe (SI-DF40P2, f = 28 kHz, Hitachi High-Tech, Tokyo, Japan).

### 2.3. Preparation and Characterization of Carboxylated CNF

Carboxylated cellulose nanofibrils were produced by TEMPO-mediated oxidation [22]. Typically, CNFs (1 g) was suspended in water (95 mL) containing TEMPO (0.016 g) and sodium bromide (0.103 g). A designed amount of the NaClO solution was added slowly to the cellulose slurry under stirring. Three TEMPO-oxidized CNFs of different carboxylate contents were prepared and labeled as TOCNFs-1, TOCNFs -2, and TOCNFs -3, which correspond to the amount of NaClO at 4.0 mmol/g, 6.0 mmol/g, and 8.0 mmol/g cellulose (dry weight), respectively. The pH of the mixture was maintained to be 10 at room temperature by adding 0.5 M NaOH, and when the pH of the mixture kept steady, the oxidation was quenched by adding ethanol (10 mL). The oxidized cellulose was washed thoroughly with water and followed by centrifugation (10000 rpm, 10 min) until no chloride ion was detectable in the supernatant by silver nitrate solution. 

The carboxylate content of the carboxylated CNF was determined by the electric conductivity titration method [23]. Carboxylated CNFs samples (3.000 g) were soaked in 0.1 M HCl solution (30 mL) for 45min, followed by adding 0.001 M NaCl (450 mL), and then the mixture was sufficiently stirred to prepare a well-dispersed slurry. Under the nitrogen atmosphere, a 0.1 M NaOH solution was added at the rate of 0.05 mL/min under stirring up to the point that the conductivity was equal to the initial. The carboxylate content of the sample was determined from the curves of conductivity and volume of NaOH consumption.

Chemical changes of CNFs before and after oxidation were analyzed by transmittance FT-IR spectroscopy using FT-IR spectrometers (TENSORII, Bruker, Ettlingen, Germany). CNFs were also subjected to crystallinity analysis using a high-resolution X-ray diffractometer (MinFLEX600, Rigaku Corporation, Tokyo, Japan). Dry CNFs films were prepared by the casting method and a 2.0 cm × 2.0 cm film was attached to the glass sample plate for the test. The scanning angle 2Ө was in steps of 0.02° at 2.5 s per step from 5° to 60°, and the CNFs crystallinity index of cellulose I was calculated according to the reported method [4].

### 2.4. Preparation and Characterization of CNFs Hydrogels

CNFs hydrogels were produced by addition of zinc chloride solution to the top of TEMPO-oxidized CNFs aqueous dispersions without stirring [12]. The TEMPO-oxidized CNFs dispersion was first homogenized by a high shear mixer (FM200A, Fluko, Shanghai, China) at 3000 rpm for 5 min, followed by degassing and transferring to a cylinder-shaped Teflon mold. A designed amount of the ZnCl_2_ solution (20 wt.%) was added dropwise along the wall of the container into the CNF dispersion without stirring. After standing for overnight, the liquid TEMPO-oxidized CNFs dispersion was transferred to a solid CNFs hydrogel. The resulting hydrogel was rinsed with water four times to remove unbounded metal ions and stored at 4 °C for analysis thereafter.

The hydrogels were subjected to compression tests using a universal testing machine (model 3367, Instron, Norwood, MA, USA). All hydrogels were cylinder shape with a diameter of 12 mm and a height of 20 mm. The compression tests were carried out at room temperature at a velocity of 1 mm/min. All engineering stress were determined to a compressive strain level of 90% and presented as an average of three individual trials [24]. The morphology of the as-prepared hydrogels was assessed by scanning electron microscopy (SEM) on PhenomPro (Phenom-World, Eindhoven, The Netherlands) at 10 kV. The specimens were first rapidly frozen using liquid nitrogen and then freeze dried for 24 h prior to SEM observation.

Swelling behavior of CNFs hydrogels was evaluated by determination of the swelling ratio. Freeze dried CNFs hydrogels were weighted (*m*_0_) and then immersed in deionized water at room temperature for 24 h. The samples were taken out from the water at selected time intervals and dried gently with blotting paper to remove excess surface water and weighed (*m*_t_). The swelling ratio was calculated by water uptake of the hydrogel (*m*_t_ − *m*_0_) divided by the dry weight (*m*_0_). Each value was averaged from three parallel measurements. 

### 2.5. Preparation of CNFs Hydrogels-Based CO_2_ Sensitive Indicator and An Application Experiment 

A CNFs hydrogel-based CO_2_ sensitive indicator was prepared by immersing 10 g CNFs hydrogel into 50 mL dye solution for 24 h. The dye solution was prepared by mixing bromothymol blue (0.04 wt.%) and methyl red (0.04 wt.%) in aqueous ethanol in a ratio of 2:3. Later on, the colored CNFs hydrogel was taken out of the dye solution and surface blotted using filter paper. Lastly, the colored CNFs hydrogel was wrapped with cling film and stored at 4 °C until use.

To conduct an application experiment on fresh-cut fruits, 500 g fresh-cut fruits were prepared and placed on a plastic tray. The CNFs hydrogel-based CO_2_ sensitive indicator was placed in the center of the tray encircled with fresh-cut fruits. Then, the package was sealed and stored at room temperature for 2 days. The color change of the CNFs hydrogel-based CO_2_ sensitive indicator was photographed each day to monitor the fruits spoilage. 

## 3. Results and Discussion

### 3.1. The CNFs Material

**Figure 1:**
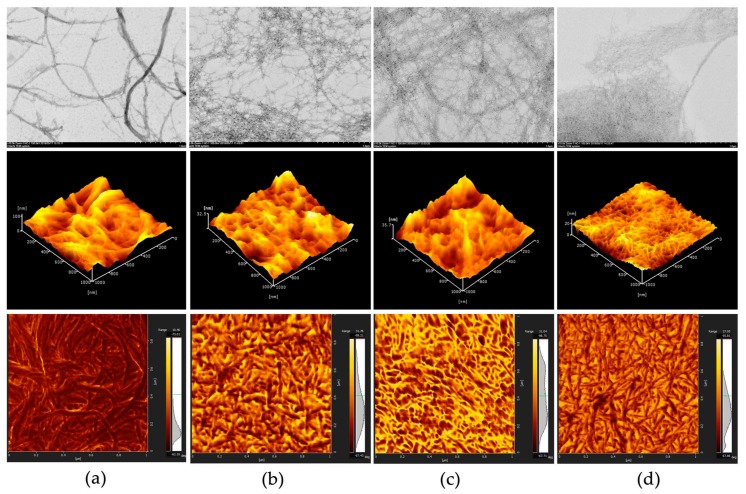
TEM image (top), AFM 3D height image (middle), and AFM phase image (bottom) of (**a**) CNFs; (**b**) TOCNF-1; (**c**) TOCNF-2; and (**d**) TOCNF-3. The scan area of AFM imaging is 1 μm × 1 μm.

In this study, CNFs was initially prepared though mechanical fibrillation of bagasse fiber/water slurries by grinder treatment and was subsequently subjected to TEMPO-mediated oxidation for generation of carboxylate groups. The morphology of CNFs and TEMPO-oxidized CNFs were identified by TEM and AFM images as shown in Figure 1. TEM observation revealed that the obtained CNFs consisted of nanofibrils with diameters in a range from 10 nm to 50 nm (Figure 1a). In contrast, after TEMPO-mediated oxidation, the sizes of CNFs were fined down and carboxylated cellulose nanofibrils (TOCNFs) of 5–10 nm wide were obtained.

AFM images in Figure 1 also indicate that CNFs 5–30 μm in length were converted to fine individual nanofibers by TEMPO-mediated oxidation. The widths of nanofibers are almost constant, whereas the lengths are varied depending on the amount of NaClO in the oxidation. It is notable that TOCNFs-3 are mostly spindle-like bundles and shorter in length compared to TOCNFs-1 and TOCNFs-2. The TOCNFs obtained under a treatment of NaClO 4 mmol/g clearly formed aggregates and still consisted of nanofibrils a few microns long. In contrast, when the amount of NaClO increased to 8 mmol/g, the TOCNFs were mostly converted to individual nanofibers with almost uniform widths and lengths. TEMPO-mediated oxidation selectively converts C6 primary hydroxyls exposed on the surfaces of crystalline cellulose microfibrils to sodium carboxylate groups. [25]. The presence of carbonate groups on the surface of CNFs may enhance the swelling of the cellulose by electrostatic repulsion to facilitate the fibrillation into individual nanofibers. Meanwhile, significant depolymerization is inevitable during the TEMPO/NaBr/NaClO oxidation of celluloses, and this depolymerization probably resulted in the downsizing of the CNFs both in width and length [26]. 

Table 1 shows that average sizes of CNFs decreased significantly after TEMPO-mediated oxidation and related to the amount of NaClO. 

When the amount of NaClO added to the TEMPO/NaBr/NaClO oxidation of CNFs at pH 10 varied from 4.0 mmol/g to 8.0 mmol/g cellulose (dry weight), the average size of obtained TOCNFs decreased from 3552 to 1044 nm. Moreover, with the increase of NaClO dosage, the carboxylate content formed on CNFs was correspondingly increased from 0.73 to 1.29 mmol/g. 

It is also noticed that the zeta potential values are dependent on carboxylate group contents on CNFs. Since the zeta potentials are directly related to the surface density of dissociated carboxyl groups, TEMPO-oxidized celluloses have more carboxylate groups on the surfaces compared to the original CNFs, resulting in increased zeta potentials. 

**Figure 2:**
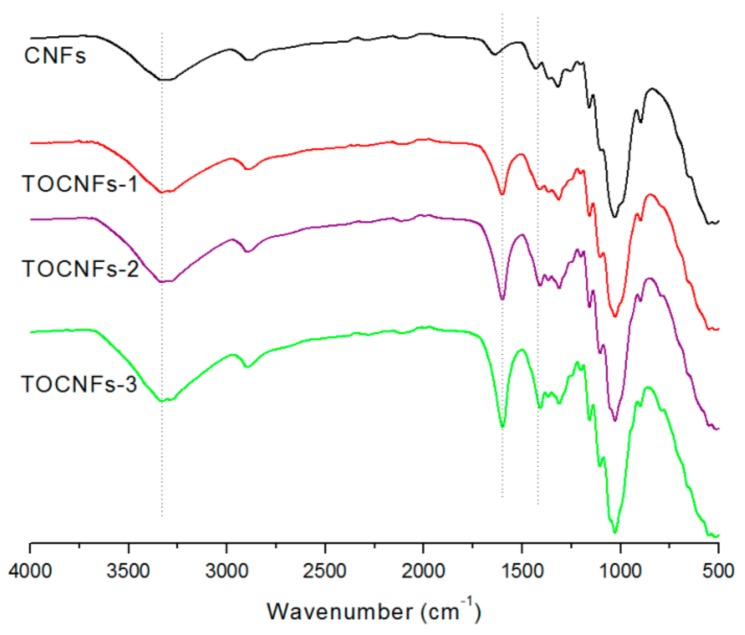
FT-IR spectra of CNFs and TEMPO-oxidized CNFs of different carboxylate contents.

The FT-IR was used to analyze changes in the chemical groups on the surface of the produced CNFs. The FT-IR spectra of the CNFs, TOCNFs-1, TOCNFs-2, and TOCNFs-3 are shown in Figure 2. For all samples, the peak observed at ~3400 cm^−1^ is attributed to O–H vibration, mainly caused by hydrogen bonds in the cellulose [27]. The characteristic strong peak at ~1600 cm^−1^ is assigned to asymmetric COO^−^ stretching vibration of carboxylate, and the increased intensity indicates that significant amounts of carboxylate groups were formed by the oxidation. Additionally, medium broad symmetric COO^−^ stretching vibration was detected from 1335 cm^−1^ up to 1440 cm^−1^, reflecting the presence of carboxylate after TEMPO-mediated oxidation [12,28].

**Figure 3:**
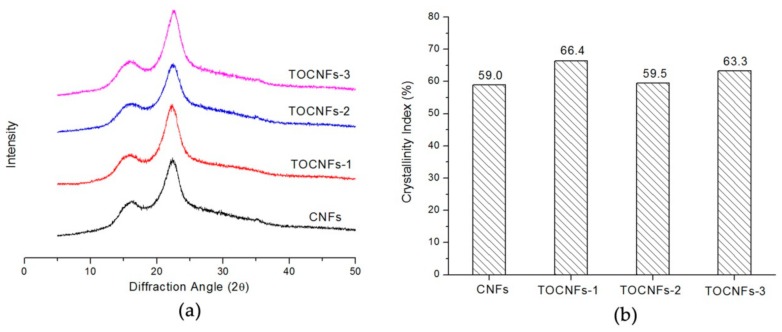
(**a**) X-ray diffraction and (**b**) crystallinity index of CNFs and TEMPO-oxidized CNFs of different carboxylate contents.

Figure 3 illustrates X-ray diffraction patterns of the CNFs before and after the TEMPO-mediated oxidation. Table 1 and Figure 2 show that significant amounts of carboxylate groups were formed in the TEMPO-oxidized CNFs. Nevertheless, the original crystal structure of cellulose I was unchanged after oxidation (Figure 3a). 

Figure 3b plots changes in crystallinity of CNFs during the TEMPO-mediated oxidation with various amounts of NaClO, with oxidation conditions correspond to those in the experiment of Figure 1 and Table 1. Crystallinity was slightly increased for the CNFs after oxidation. The slight increase in crystallinity is probably due to partial loss of the disordered regions during the oxidation because of their increased water-solubility. Since bagasse CNFs contain low-crystalline cellulose and some hydrolyzed disordered regions during grinding, the oxidation selectively takes place in these accessible or disordered regions, resulting in a slight increase in crystallinity. Similar results were observed in steam-explosion of native cellulose samples as well as cellulose/ammonia pretreated cellulose cotton linter after TEMPO-mediated oxidation [22].

Overall, the TEMPO-mediated oxidation led to significant changes in characteristics of CNFs, such as imparting sufficient carboxylate groups on CNFs surfaces to be cross-linked with Zn^2+^ for gelation, disintegrating CNFs into individual fibrils of appropriate sizes to be served as structural units for gel network.

### 3.2. Formation of CNFs Hydrogels

**Figure 4:**
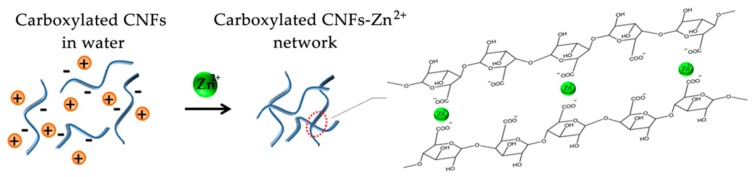
Schematic illustration of carboxylated CNFs crosslinking with Zn^2+^ for gelation.

It is interesting to note that CNFs hydrogels are unable to be produced by adding Zn^2+^ to the original CNFs, irrespective of the addition amounts of Zn^2+^ and initial concentration of CNFs. In contrast, CNFs hydrogels were produced by diffusing Zn^2+^ into the TOCNFs dispersion. It is believed that the binding affinity of metal cations with carboxylate groups initiates the gelation process [12]. According to the scheme shown in Figure 4, the C6 carboxylate groups of cellulose are expected to be cross-linked with Zn^2+^ for gelation [29].

Initially, TOCNFs were present as anionically charged individual fibrils in water due to the large amounts of sodium carboxylate groups present on the TOCNFs surfaces [25]. After adding in Zn^2+^ solution, the inter-fibril electrostatic repulsive forces generated by the TOCNFs carboxylate surface charges have been screened, bringing fibrils to close and reversing the repulsive forces to attractive cohesive forces [10]. The metal-carboxylate interactions are expected to form on the surface of the same fibrils or adjacent fibrils, leading to the interwoven fibrous networks and finally the formation of the CNFs hydrogel. 

Besides, there is no structural change to CNFs themselves by Zn^2+^ and the mechanical properties of the gels are believed to be related to the inter-fibril attractive interactions. In this regard, it is proposed that carboxylate content, concentrations of CNFs, and Zn^2+^ may directly influence the degree of gelation and mechanical strength of corresponding hydrogels.

### 3.3. Mechanical Strength of Hydrogels

**Figure 5:**
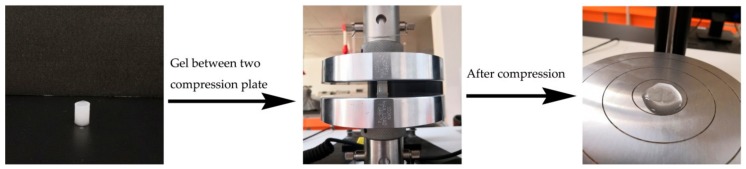
CNFs hydrogel for compression testing.

The prepared CNFs hydrogels were free-standing and translucent. Figure 5 shows the self-supporting form of the gels and a corresponding experiment set-up for the compression test. Cylinder-shaped hydrogels with a diameter of 12 mm and a height of 20 mm were prepared using a Teflon mold.

There are several reports about the behavior of hydrogels in compression and most measurements have commonly used engineering stress versus engineering strain behavior [11,30,31]. Engineering stress is defined as the applied load divided by the initial cross-sectional area of the specimen. In fact, hydrogels contain a high amount of water and have substantial deformations during compression. In this case, the cross-sectional area of the specimen constantly changes during compression and the true stress is not measured directly in the test [32,33]. Subject to specific situations, dynamic mechanical analysis or modified version of the Watts and Ford techniques were suggested to use in the determination of the compression behavior of hydrogel samples [11,33]. Therefore, it would be prudent to conduct the compression test in this study and comment on the CNFs hydrogel compressive performance based on the relevant stress–strain results. 

The orthogonal design is an effective method for optimizing a research that involves multiple variables [34]. TONC concentration, the content of carboxylate groups on CNFs, and concentration of zinc ions were selected as the three experimental factors of orthogonal tests, and each factor had three levels. It was assumed that no two factors interacted with each other. An orthogonal array table L9(3^3^) was used, and the test program is given in Table 2. 

In order of decreasing effect on the compressive stress of hydrogels, the variables were TOCNFs concentration > content of carboxylate groups > concentration of zinc ions. The optimum TOCNFs concentration required to induce stiff gelation was 3.0 wt.%.; a lower TOCNFs concentration was insufficient for the formation of strong CNFs hydrogels even at a high carboxylate groups content. It was hypothesized that the gelation process occurs between two or three fibrils. The binding interactions of fibrils at a high TOCNFs concentration are of great opportunity to form a strong network, resulting in a gel structure with high compressive strength. From the results, it can be concluded that the increase of TOCNFs concentration improves the mechanical properties. However, further increase TOCNFs concentration over 3 wt.% will lead to translucent reduction as well as cost increase of the hydrogel. The TOCNFs hydrogel prepared at 3.0 wt.% is feasible to maintain shape integrity for load-bearing situations in the following intelligent packaging application experiment. Therefore, the optimum TOCNFs concentration was 3.0 wt.% and no higher concentrations were checked in this study. Besides, carboxylate groups were reactive sites for the gelation, and high carboxylate group contents may have a high tendency to cross-link between two nanofibrils, considering the electrostatic attraction and geometrical needs to interact with surrounding Zn^2+^. The optimal carboxylate group content in this study was 1.29 mmol/g. In contrast, the concentration of zinc ions had little influence on the strength of hydrogels, even though high concentrations of Zn^2+^ have a higher tendency than diluted Zn^2+^ to cross-link between adjacent fibrils. 

In sum, the mechanical properties of gels were primarily influenced by the TOCNFs concentration, followed by the content of carboxylate groups and concentration of zinc ions. The best conditions included a TOCNFs concentration of 3.0 wt.%, carboxylate group content of 1.29 mmol/g, and zinc ions concentration of 0.2mol/l. 

**Figure 6:**
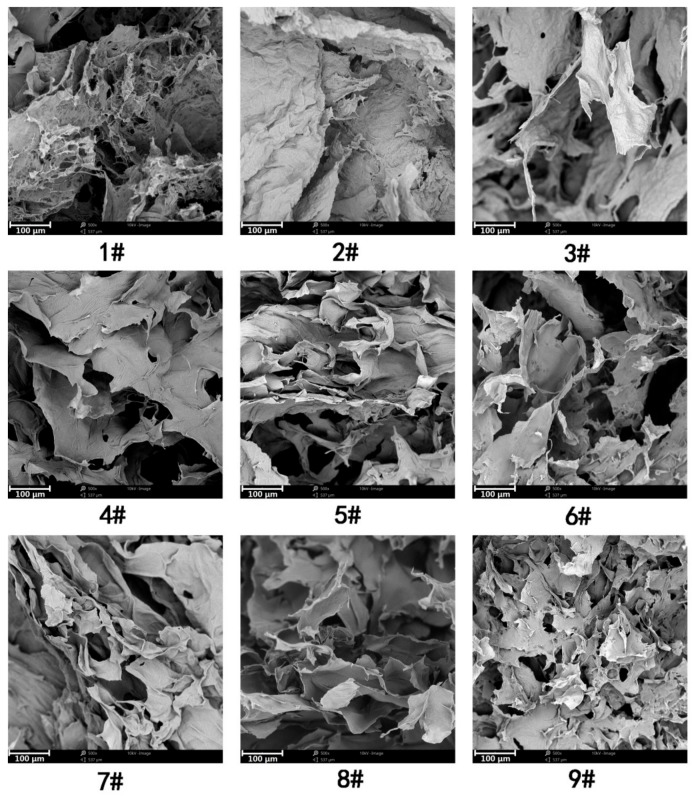
SEM micrographs of the freeze-dried CNFs hydrogels corresponding to those in the experiment of Table 2.

The hydrogels can be freeze-dried into sponge-like aerogels and the structures were investigated by SEM, as shown in Figure 6. Significant differences in the network structures are observed between gels prepared using TOCNFs of different concentration and carboxylate group content at different concentrations of zinc ions. 

For hydrogels prepared at 1.0 wt.% TOCNFs concentration, the obtained aerogel barely contains pore structures, but contiguous dense films as presented in Figure 6 (sample #1, #2, and #3). These dense TOCNFs film structures may exclude more water, leading to the volume shrinkage as observed in the SEM images. When the TOCNFs concentration increased to 2.0 wt.% (sample #4, #5, and #6), the TOCNFs hydrogel networks were still relatively non-uniform at the macroscopic level and heterogeneous with dense film regions. The increase of TOCNFs concentration to 3.0 wt.% leads to visible pores in the SEM images and the morphology is more foam-like. A fine network of interconnected nanofibrils with open pore structures surrounded by thin films was observed for gels prepared at the optimum condition (sample #9). Furthermore, the compressive stresses of the gel increase to 337.16 kPa at 90% strain, which is significantly higher than the values of other hydrogels. It was believed that TOCNFs with multiple carboxylate groups are easily bridged together by Zn^2+^ gelation at relatively high concentrations. Additionally, the large number of hydroxyl and carboxylate groups is sufficient to form strong hydrogen bonding to allow a great inter-fibril association. Besides, the interwoven CNFs network is expected to alleviate stress concentration and results in a strong gel structure with a high compressive strength [18].

Overall, the results presented above suggest that the mechanical strength of the CNFs hydrogel is easily adjustable by controlling concentrations of zinc ions and TOCNFs in dispersion systems, as well as the content of carboxylate groups on TOCNFs, and present an interesting way of tailoring CNFs hydrogel for specific applications.

**Figure 7:**
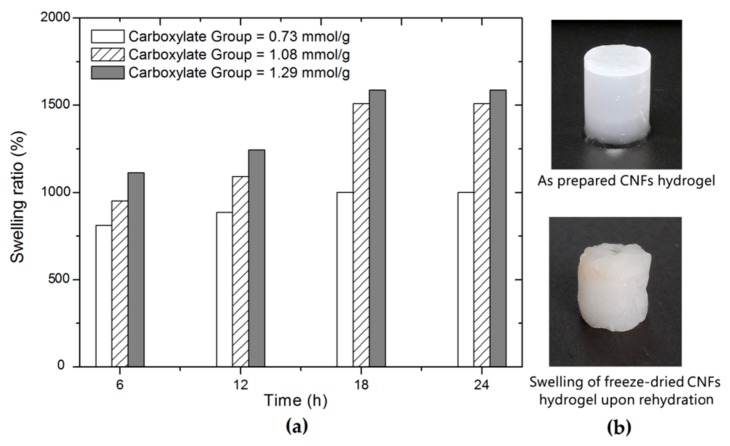
(**a**) Swelling ratio of CNFs hydrogels at a different soaking time; (**b**) visual appearance of a CNFs hydrogel prepared with TOCNFs of carboxylate group 1.29 mmol/g. The representative hydrogels were prepared at a fixed TOCNFs concentration of 3.0 wt.% and Zn^2+^ concentration of 0.2 mol/L.

The swelling ratio of a hydrogel is used for the evaluation of the water uptake capacity [35]. It was reported that the water retention ability of CNFs increased with increasing the carboxylate content [22]. Therefore, the content of carboxylate groups probably impact on swelling behaviors of corresponding CNFs hydrogels. The CNFs hydrogels with different carboxylate groups were prepared at a fixed TOCNFs concentration of 3.0 wt.% and zinc ions concentration of 0.2 mol/L, which is the optimum condition for gel formation as previously mentioned. The swelling properties of CNFs hydrogels as a function of time are shown in Figure 7. 

It was found that the swelling ratio was above 800% for all samples after 6 h of immersion and changed with time. The swelling ratio of samples increases quickly in the first 18 h and then kept steady until 24 h of immersion. Increasing the content of carboxylate groups of the CNFs leads to a significant change in the swelling ratio. It was observed that the hydrogel containing the content of carboxylate groups 1.29 mmol/g exhibited the highest ability to retain water (15,800% after 24 h of soaking). The increase in the swelling ratio was presumably due to the increased carboxylate groups that favor water retention during rehydration. In addition, the CNFs hydrogel prepared at relatively high carboxylate groups tends to have a fine network after freeze drying as evidenced by the SEM images (Figure 6). This fine network with open pore structures was assumed to contribute to water retention during rehydration. Interestingly, an obvious geometric change and translucence reduction are also observed for freeze-dried hydrogel after 24 h soaking in water relative to the initial hydrogel. The removal of water from the hydrogel may cause the hornification of CNFs as well as the collapse of pore structure [36], and thus probably result in the deteriorated performance of the restored hydrogel after rehydration. In this case, it is preferable to keep the hydrogel in wet states for suitable applications.

### 3.4. Application of Hydrogels

Fresh-cut fruits and vegetables are highly perishable due to potential microbial spoilage. Carbon dioxide is a common by-product in food spoilage, and therefore simply detection of CO_2_ levels in the package is an ideal way to know the freshness of the packed food [37]. Intelligent packaging is a practical technology in food packaging that is capable of sensing and providing information about the functions and properties of the packaged products. The CO_2_ sensitive indicator is a typical type of freshness indicators for the intelligent packaging applications. In particular, color-based CO_2_ sensitive indicators have been used for the real-time determination of freshness of various food products, such as packaged chicken breast [38], intermediate-moisture dessert [39], and fresh-cut bell peppers [40].

**Figure 8:**
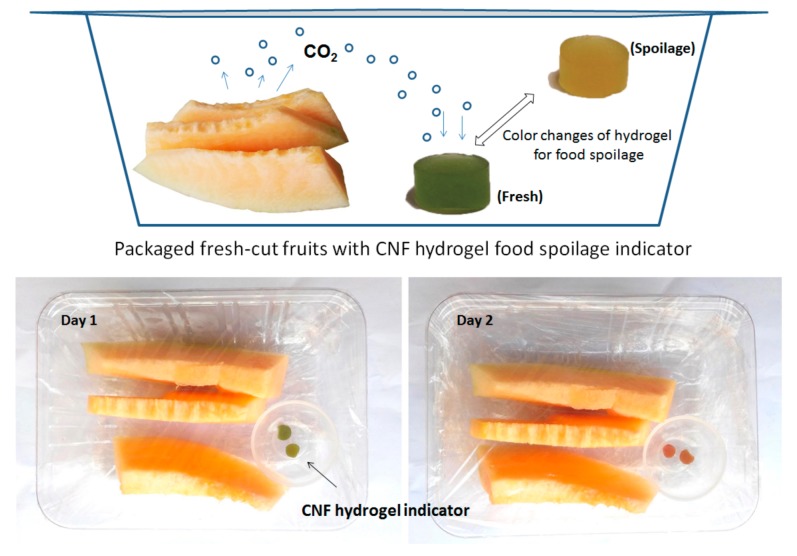
Scheme of colored CNFs hydrogels for intelligent food packaging applications.

Most color-based CO_2_ sensitive indicators are reliant upon the change in pH which happens when CO_2_ dissolves in water. The detection process involves a color change of a pH sensitive dye, which reacts with the protons generated CO_2_ in water [41]. The presence of water or moisture is essential to the work of such indicators; however, most color-based CO_2_ sensitive indicators are in forms of solid dry plastic films or paper strips [38,39,40]. In fact, hydrogels are regarded as a suitable material for CO_2_ sensor due to the fact that it contains abundant water and offers fast protons generation in response to external CO_2_ stimuli. Thus, in this paper, we prepared a CNFs hydrogel-based CO_2_ sensitive indicator and explored its potential application in intelligent packaging. Figure 8 illustrates packed fresh-cut fruits together with a CNFs hydrogel-based CO_2_ sensitive indicator in a plastic container that was tightly sealed.

As shown in Figure 8, a brief study of a CNFs hydrogel-based indicator exposed to fresh-cut fruits revealed a clear change of color with food freshness. The colored CNFs hydrogel indicator was initially dark green with no remarkable change when the fresh-cut fruits were fresh. However, a clear color change from dark green to orange yellow was observed at day two, indicating a high level generation of CO_2_ due to micro-organisms proliferation and associated food spoilage. In addition, the abundant water contents in this CNFs hydrogel indicator can facilitate the quick CO_2_ dissolves and protons generation, offering a fast response time and robust reproducibility. Additional works in respect of the effect of temperature, limit CO_2_ levels of detection, and color persistence should be taken to fabricate a robust CNFs hydrogel-based indicator for use in intelligent packaging. 

## 4. Conclusions

A self-supporting cellulose hydrogel was prepared from bagasse CNFs using the Zn^2+^-mediated cross-linking method. TEMPO process was used to generate negatively charged surface carboxylate groups on CNFs surface to provide a high binding capability to Zn^2+^. Three TEMPO-oxidized CNFs of different carboxylate contents were prepared and characterized. TEM and AFM microscopes suggested that the sizes of CNFs were fined down and carboxylated cellulose nanofibrils (TOCNFs) of 5–10 nm wide, 200–500 nm long, carboxylate contents 0.73–1.29 mmol/g were obtained. The final structures and compressive strength of hydrogels are primarily influenced by interfibril Zn^2+^-carboxylate interactions, following the order of TOCNFs concentration > content of carboxylate groups > concentration of zinc ions. It was demonstrated that the strength of the hydrogels can be tuned by choice of TOCNFs and this bagasse derived CNFs hydrogel can be fabricated to CO_2_ sensitive indicators for use in intelligent food packaging. 

## Figures and Tables

**Table 1:** Characteristics of cellulose nanofibrils before and after TEMPO oxidation.

Sample	Average Size (nm)	Zeta Potential (mv)	Carboxylate Group Content (mmol/g)
CNFs	3705	−20.7	0.12
TOCNFs-1	3552	−41	0.73
TOCNFs-2	2673	−43	1.08
TOCNFs-3	1044	−51.8	1.29

**Table 2:** Orthogonal experiment for CNFs hydrogels preparation.

Sample	TOCNFs Concentration (wt.%)	Carboxylate Group Content (mmol/g)	Zn^2+^ Concentration (mol/L)	Compressive Stress (kPa)
1	1.0	0.73	0.1	46.63
2	1.0	1.08	0.2	76.35
3	1.0	1.29	0.3	76.61
4	2.0	0.73	0.2	156.27
5	2.0	1.08	0.3	183.46
6	2.0	1.29	0.1	170.14
7	3.0	0.73	0.3	226.73
8	3.0	1.08	0.1	253.49
9	3.0	1.29	0.2	337.16
K1 ^1^	66.53	143.21	156.75	
K2	169.96	171.10	189.93	
K3	272.46	194.64	162.27	
*R* ^2^	205.93	54.43	33.18	

^1^*K* is an average value for each parameter based on the levels; *R* is the difference of the maximum and minimum value of K, representing impact order on the experiment.
